# Atypical hemolytic uremic syndrome associated with pregnancy: A case report

**DOI:** 10.3892/mi.2025.270

**Published:** 2025-09-23

**Authors:** Karen Paola Quintana Barragán, Hugo Alberto Roblero López, Luisa Fernanda Montemayor Burrola, América Villalobos Ulate, Ruben Alejandro Almela Mendoza, José Ángel Sánchez Ochoa

**Affiliations:** Department of Internal Medicine, Hospital General Regional No. 1, Mexican Institute of Social Security, Chihuahua, Chihuahua 31000, Mexico

**Keywords:** thrombotic microangiopathy, hemolytic uremic syndrome, pregnancy

## Abstract

Hemolytic uremic syndrome (HUS) is a microangiopathy characterized by hemolytic anemia, thrombocytopenia and acute renal failure. It affects 2 to 3 individuals per 100,000 in the population, with a higher prevalence among adult women. HUS is caused by the abnormal activation of the complement system, leading to endot2helial damage and the formation of microthrombi in renal capillaries, which determines the severity of the condition. Pregnancy may predispose individuals to HUS due to changes in the maternal immune system. The present study describes the case of a 29-year-old female patient who presented postpartum with purulent discharge, severe anemia (hemoglobin level, 4.6 g/dl), thrombocytopenia, acute kidney injury (creatinine level, 10.6 mg/dl) and elevated levels of lactate dehydrogenase (1,450 U/l). Despite antibiotic therapy and blood transfusions, she developed anuria, metabolic acidosis and acute pulmonary edema, requiring admission to the intensive care unit and mechanical ventilation. A peripheral blood smear revealed >10 schistocytes per high-power field. A kidney biopsy confirmed HUS. Following stabilization with plasma infusions, corticosteroids and supportive care, she was discharged with intermittent hemodialysis and continues under nephrology follow-up. On the whole, HUS is a rare condition that is increasingly recognized. The case presented herein highlights the rapid clinical progression of this condition, emphasizing the importance of diagnostic suspicion to reduce mortality and improve prognosis.

## Introduction

Hemolytic uremic syndrome (HUS) is a thrombotic microangiopathy (TMA) characterized by the triad of microangiopathic hemolytic anemia, thrombocytopenia and acute kidney injury (1). HUS is classified into two main types: i) Typical HUS, most commonly caused by Shiga toxin-producing *Escherichia coli* infections; and ii) atypical HUS (aHUS), which is primarily related to the dysregulation of the complement system due to genetic mutations. Typical HUS mainly affects children, whereas aHUS is more frequent among adults, particularly women during pregnancy or postpartum. Distinguishing between these forms is essential for an accurate diagnosis and management (2,3). First documented by Gasser *et al* (4) in 1955, HUS primarily affects the renal vasculature, resulting in arteriolar fibrinoid necrosis (5). Its global incidence is ~2 to 3 cases per 100,000 individuals annually (6). While typical HUS affects children of both sexes equally, aHUS is more frequently diagnosed in adult women, potentially linked to pregnancy-related triggers (7). As aforementioned, the syndrome is categorized into two forms: Typical HUS, commonly associated with infections such as Shiga toxin-producing *Escherichia coli* (STEC), and aHUS, often driven by genetic mutations affecting the complement system (8). Both variants arise from the dysregulation of the alternative complement pathway, leading to the formation of the membrane attack complex (C5b-9) (9). This process damages endothelial cells and promotes a procoagulant state, inducing platelet deposition and resulting in microthrombi within the renal microvasculature that determines disease severity (10).

Clinically, HUS presents with fatigue, pallor due to anemia and lethargy, progressing to renal manifestations, including hematuria, proteinuria, and, in severe cases, anuria (11). Extrarenal complications, such as cardiac and neurological involvement, occur in up to 20% of patients (12,13). Diagnosis is established through laboratory findings, such as elevated levels of lactate dehydrogenase, schistocytes, thrombocytopenia and renal impairment, supported by imaging to assess kidney involvement (14). A histological evaluation may be required to differentiate HUS from other TMAs, such as thrombotic thrombocytopenic purpura (15,16). Treatment is tailored to disease severity and recurrence risk (17), encompassing supportive care (e.g., hydration and transfusions), immunosuppressive therapy (18), and, in refractory cases, renal replacement therapy or kidney transplantation. Genetic testing is recommended, as ~50% of aHUS cases are associated with mutations in complement-regulating genes, notably complement factor H (CFH) (19-21). Additional risk factors include certain medications, autoimmune disorders and chronic illnesses (22,23).

Pregnancy is a recognized risk factor for aHUS, likely due to the decreased production of complement regulatory proteins and altered maternal immunity (24). However, diagnosis in the postpartum period is challenging, as its features overlap with obstetric conditions such as preeclampsia and hemolysis, elevated liver enzymes and low platelets (HELLP) syndrome, often delaying appropriate management (25,26). Given the rarity of aHUS and its potential for severe morbidity and mortality, the present study describes the case of a 29-year-old female patient who developed aHUS 4 days following a cesarean delivery.

## Case report

### Initial presentation

A 29-year-old female patient (G2P1C1) presented to the Emergency Department at the Gynecology and Obstetrics Hospital No. 15 of the Mexican Social Security Institute (Chihuahua, Mexico) on the 4th day postpartum following a cesarean section, reporting purulent discharge from the vaginal and surgical sites, along with urinary symptoms. The patient described a 4-day history of fever (38.9˚C), asthenia, fatigue, dyspnea and pallor. Her medical history was notable for a cesarean section at 39 weeks of gestation, complicated by an estimated 3-liter obstetric hemorrhage.

Upon admission, laboratory tests revealed severe normocytic normochromic anemia (hemoglobin level, 4.6 g/dl) and acute kidney injury (creatinine, 10.6 mg/dl; urea, 286 mg/dl; blood urea nitrogen, 133.47 mg/dl); urinalysis revealed leukocyturia (20-22 leukocytes per field) with granular casts. The patient received empirical intravenous antibiotics and 3 units of packed red blood cells before being transferred to a secondary care facility the Regional General Hospital No. 1 of the Mexican Social Security Institute, Morelos, Mexico for further management.

### Diagnostic workup

The nephrology service evaluation identified anuria (urine output 0.08 ml/kg/h), metabolic acidosis, azotemia and hyperkalemia, necessitating transfer to the intensive care unit (ICU) for intermittent hemodialysis. Initially, acute tubular necrosis secondary to ischemic injury from obstetric hemorrhage was suspected. A wound culture was obtained and dual antibiotic therapy was initiated. The general surgery team drained 20 ml of purulent fluid from the surgical site, resulting in transient clinical improvement.

After 3 days, the patient developed lower respiratory symptoms requiring supplemental oxygen. A chest computed tomography scan revealed bilateral hilar reticular opacities. Given these findings, and considering it was peak influenza season, empirical treatment with oseltamivir (75 mg, administered orally twice daily) was initiated following the institutional protocol (27). However, subsequent testing ruled out influenza infection.

### Treatment course

The patient subsequently exhibited signs of fluid overload and acute pulmonary edema, which was refractory to non-invasive ventilation, leading to 4 days of mechanical ventilation.

Due to persistent severe anemia, thrombocytopenia and laboratory evidence of hemolysis (schistocytes on blood smear, elevated lactate dehydrogenase without hyperbilirubinemia), a peripheral blood smear was performed, revealing >10 schistocytes per high-power field. She received fresh frozen plasma and intravenous corticosteroids. Thrombotic microangiopathy was investigated; however, complement levels were normal and autoantibody tests yielded negative results: ANCA, 0.8 ng/m; anti-dsDNA, 9.3 IU/ml; C3, 107 mg/dl; C4, 24.8 mg/dl; ADAMTS13 activity, 70%; and anticardiolipin, 1.2; these findings ruled out thrombotic thrombocytopenic purpura.

**Figure 1:**
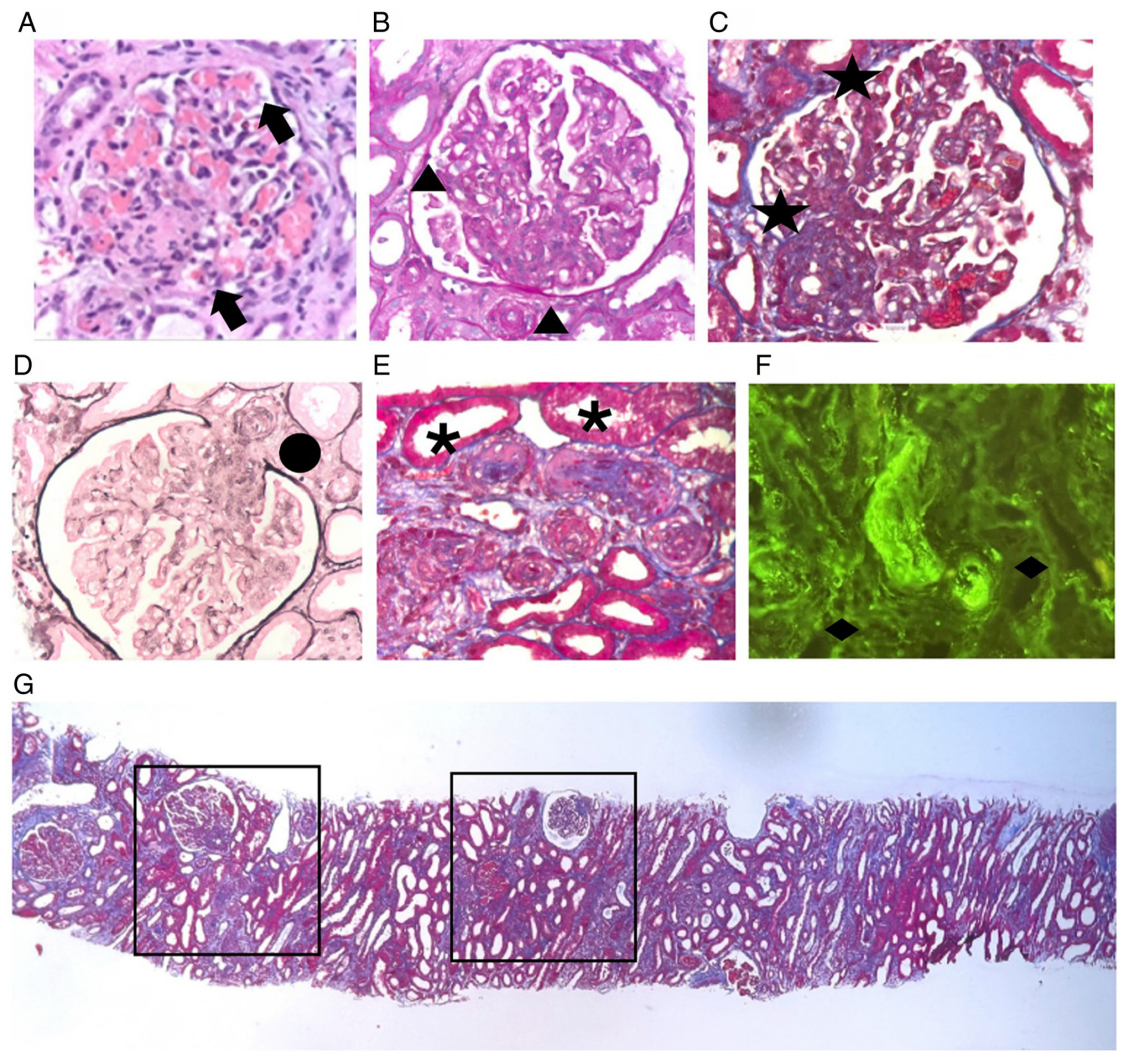
Kidney biopsy findings consistent with thrombotic microangiopathy (magnification, x40). (A) Glomerulus with mesangiolysis, endothelial swelling and segmental duplication of the glomerular basement membrane (H&E staining). (B) Glomerular capillary loops illustrating mesangial expansion and occlusion of capillary lumina by proliferating mesangial and endothelial cells (PAS staining). (C) Extensive thrombotic occlusion of glomerular capillaries and mesangial matrix expansion (Masson’s trichrome staining). (D) Duplication of the glomerular basement membrane and collapsed capillary tufts suggestive of chronic microangiopathy (Jones silver staining). (E) Arteriolar thrombosis and marked intimal thickening of interlobular arteries (Masson’s trichrome staining). (F) Immunofluorescence showing positive staining for fibrinogen (2+) within mesangium, endothelium, and arterial thrombi. (G) Low-power panoramic view of the renal cortex illustrating widespread vascular changes, tubular atrophy (~10%), interstitial fibrosis, and vascular remodeling consistent with arteriolonephrosclerosis and ‘onion-skin’ appearance of small arteries (Masson’s trichrome staining). These histopathologic findings are characteristic of thrombotic microangiopathy in both acute and chronic phases, with the absence of immune complex deposition confirming a non-immune-mediated etiology. Black arrows indicate endothelialitis and mesangiolysis. Black triangles indicate capillary lumen obstruction by cell proliferation. Black stars indicate fibrin thrombi in the glomerulus. Black circle indicates glomerular basement membrane duplication (membranoproliferative pattern). Black asterisk symbols indicate arterial thrombus with subintimal fibrosis. Black rhombus symbols indicate fibrinogen deposition (+2). Black squares indicate arteriolonephrosclerosis with an ‘onion-skin’ pattern.

Given the ongoing hemolytic anemia, renal impairment and the absence of positive autoantibodies, a percutaneous kidney biopsy was performed. Renal tissue samples were fixed in 10% neutral buffered formalin for 24 h at room temperature, embedded in paraffin, and cut into 4-µm-thick sections. Hematoxylin and eosin (H&E), periodic acid-Schiff (PAS), Masson's trichrome and Jones silver staining were performed following standard histopathological protocols using reagents supplied by Sigma-Aldrich^®^ (Merck KGaA). The sections were stained at room temperature according to the manufacturer's recommendations (H&E, hematoxylin 10 min, eosin 3 min; PAS, periodic acid 5 min, Schiff's reagent 15 min; Masson's trichrome: Sequential staining was performed with Weigert's iron hematoxylin, Biebrich scarlet-acid fuchsin, phosphotungstic/phosphomolybdic acid and aniline blue; Jones silver: Methenamine silver impregnation with periodic acid oxidation). Microscopic examination and image acquisition were performed using a Nikon Eclipse E200 microscope^®^ (Nikon Corporation). A histopathological analysis demonstrated thrombotic microangiopathy in both acute and chronic phases, with glomerular and interlobular artery thrombosis, membranoproliferative glomerulonephritis, diffuse endothelialitis without immune complex deposition and severe arteriolonephrosclerosis with onion-skin elastosis. Later, the sections were incubated overnight at 4˚C with the following primary antibodies: IgA (1:100; cat. no. A0262), IgG (1:200; cat. no. A0423), IgM (1:100; cat. no. A0425), C1q (1:100; cat. no. A0136), C3c (1:200; cat. no. A0062), fibrinogen (1:100; cat. no. A0080), kappa light chain (1:200; cat. no. A0191), lambda light chain (1:200; cat. no. A0193) and albumin (1:200; cat. no. A0001) (all from Dako, Agilent Technologies, Inc.). After washing, the slides were incubated with horseradish peroxidase (HRP)-conjugated secondary antibodies [goat anti-rabbit IgG (cat. no. P0448) or goat anti-mouse IgG (cat. no. P0447) (both from Dako, Agilent Technologies, Inc.), depending on the primary antibody] for 1 h at room temperature. Visualization was achieved using 3,3'-diaminobenzidine (DAB) substrate solution (cat. no. K3468; Dako, Agilent Technologies, Inc.). Immunohistochemical staining for immunoglobulins (IgA, IgG and IgM), complement components (C1q and C3c), fibrinogen, kappa, lambda and albumin was uniformly negative, confirming the diagnosis of HUS (Fig. 1).

### Clinical outcomes

**Figure 2:**
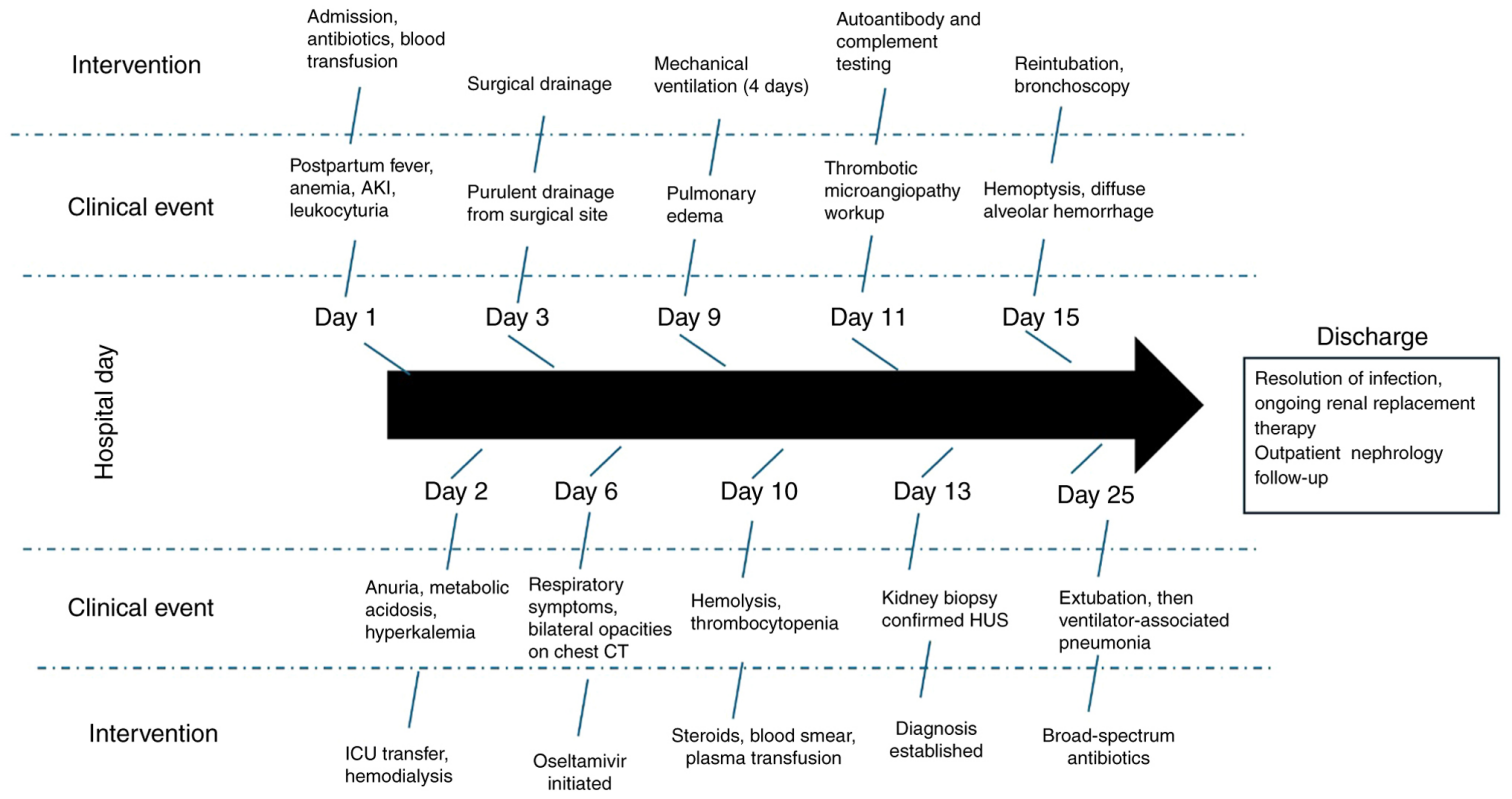
Clinical timeline and diagnostic approach in a postpartum patient with atypical HUS. AKI, acute kidney injury; CT, computed tomography HUS, hemolytic uremic syndrome.

Several days later, the patient developed exsanguinating hemoptysis. Bronchoscopy revealed diffuse alveolar hemorrhage without specific lesions, leading to a diagnosis of diffuse alveolar hemorrhage. However, no biopsy was performed, as no specific lesions were visualized. The patient required reintubation on hospital day 15 and was successfully extubated after 10 days. However, she developed ventilator-associated pneumonia, necessitating broad-spectrum antibiotics, which delayed the initiation of immunosuppressive therapy. The rapid postpartum onset of anemia, thrombocytopenia, and acute kidney injury in the patient described herein closely mirrors the presentation described in previously reported cases of pregnancy-associated aHUS, in which early complement blockade has often been linked to improved hematologic recovery, although renal outcomes remain variable. A distinctive feature in the present case was the presence of multiple severe complications, including diffuse alveolar hemorrhage and ventilator-associated pneumonia, which are less frequently described in the literature and may have contributed to delays in targeted treatment. Following improvement and resolution of the infection, she was discharged with ongoing intermittent hemodialysis and remains under nephrology follow-up for renal replacement therapy. Renal transplantation from a first-degree relative (sister) was performed following renal replacement therapy, with preserved graft function on follow-up. A concise summary of key laboratory parameters at major clinical milestones is presented in Table I. The chronological sequence of clinical events and interventions is depicted in Fig. 2.

## Discussion

The present case report details a young female patient who, on the 4th day postpartum following a cesarean section, experienced an acute onset of hemolytic anemia, thrombocytopenia, significant hemorrhage and acute kidney injury, culminating in a diagnosis of pregnancy-associated HUS. HUS, a rare TMA. It has an estimated global incidence of 2 to 3 cases per 100,000 individuals annually, and it predominantly affects children and young adults, with incidence peaks at ages 21 and 25 uears for males and females, respectively (28).

TMAs are categorized by their underlying mechanisms: Primary TMAs, such as thrombotic thrombocytopenic purpura (TTP) and typical HUS (HUS-Tx), stem from intrinsic genetic defects, whereas secondary TMAs, including aHUS, are precipitated by infections, autoimmune conditions, or other triggers such as aHUS or microangiopathic HUS (29). Although rare, HUS can manifest during pregnancy or the immediate postpartum period, particularly in genetically susceptible young women (30). Typically emerging within the first few days postpartum, it affects ~1 in 25,000 pregnancies. Despite its rarity, HUS carries severe implications, with up to 70% of affected women requiring long-term renal replacement therapy, underscoring the need for prompt recognition to improve prognosis (31).

The identification of HUS is challenging, as its differential diagnosis includes all forms of TMAs, (both those directly and indirectly mediated by the complement system), as well as common pathologies that present with similar clinical manifestations (5). The patient in the case described herein presented a clinical picture consistent with various hemolytic disorders associated with pregnancy and the puerperium. Additionally, the history of significant bleeding during the obstetric event is particularly noteworthy, as previous reports have suggested that blood loss >1,500 ml in women without a prior pathological history may indicate a diagnosis of HUS (32).

Acute anemia is a frequent finding in postpartum HUS and presents a broad differential etiology, often delaying initial diagnostic suspicion (33). Renal manifestations, including azotemia and electrolyte imbalances, aligned with prior descriptions, yet necessitated the exclusion of more common entities such as preeclampsia, HELLP syndrome and hypovolemic acute kidney injury through systematic evaluation (34).

This protocol included, for example, the identification of abundant schistocytes on the blood smear, which suggests microangiopathy, and an elevated level of lactate dehydrogenase, indicative of prolonged cellular lysis (35). These findings, together with other laboratory results, clinical signs of cellular ischemia, refractory anemia and worsening symptoms, strongly supported the suspicion of microangiopathy.

For this reason, the analyses of antibodies and complement fractions were performed (36). To determine the triggering cause, the use of the ADAMTS13 test via fluorometric assay was also warranted (37). After evaluating the results, the diagnosis of the patient was ultimately determined. Additionally, it was found that complement levels were within normal ranges (C3, 107 mg/dl; C4, 24.8 mg/dl) and ADAMTS13 activity was 70%, effectively ruling out thrombotic thrombocytopenic purpura. aHUS has a strong genetic basis, with mutations commonly identified in complement regulatory genes, such as CFH, CFI, MCP, C3 and deletions in CFHR1/3. Genetic testing is critical for confirming diagnosis, assessing prognosis and guiding family counseling. However, genetic analysis was not performed in the present study due to limited availability at the Gynecology and Obstetrics Hospital No. 15 of the Mexican Social Security Institute. Expanding access to such testing in the future is vital for optimizing patient management and understanding disease pathogenesis. Given the overlapping clinical features, distinguishing aHUS from other pregnancy-associated thrombotic microangiopathies is crucial. HELLP syndrome typically presents with hemolysis, elevated liver enzymes and low platelets before or shortly after delivery, often associated with hypertension and proteinuria, both of which were absent in the patient described herein. Although severe preeclampsia is characterized by hypertension and signs of organ dysfunction, the patient in the present study had no hypertensive episodes or proteinuria throughout her pregnancy or postpartum course. TTP is usually marked by severe ADAMTS13 deficiency (<10%), neurological symptoms and minor renal involvement; by contrast, the ADAMTS13 activity of the patient in the present study was within normal limits (70%) and acute kidney injury was a prominent feature.

The constellation of findings, postpartum anemia, thrombocytopenia, acute kidney injury, normal ADAMTS13 activity and confirmation of thrombotic microangiopathy on kidney biopsy, strongly supports the diagnosis of pregnancy-associated aHUS, effectively distinguishing it from other differential diagnoses.

However, multiple complications arose at that time, extending her treatment and duration of hospitalization and necessitating subsequent outpatient follow-up while she continued renal replacement therapy. Eculizumab was considered as a therapeutic option; however, it was not administered due to the presence of active infectious complications, including sepsis and ventilator-associated pneumonia, which contraindicated its use in this setting. Current treatment options for aHUS include plasma exchange or infusion, immunosuppressive therapies, such as corticosteroids and complement inhibitors such as eculizumab (38). Eculizumab has exhibited notable efficacy by inhibiting terminal complement activation, reducing disease progression, and improving survival rates (39). However, its use may be contraindicated in patients with active infections, as was the case here, and it is also limited by availability and cost. Plasma exchange remains a mainstay therapy when eculizumab is unavailable or unsuitable, although critical illness may restrict its feasibility (40,41). Additionally, there were institutional and resource limitations regarding its availability. As regards plasma therapy, the patient received fresh frozen plasma infusions and intravenous corticosteroids, which led to temporary stabilization. Plasmapheresis (plasma exchange) was considered but ultimately not initiated, primarily due to the patient's critical hemodynamic status and the decision of the multidisciplinary team prioritizing infection control and respiratory management.

The present case report highlights the rapid clinical progression of a rare condition and emphasizes the importance of maintaining a high index of suspicion in women presenting with similar characteristics to facilitate prompt treatment. This case shares a number of clinical and laboratory features with previously reported cases of pregnancy-associated aHUS, including rapid postpartum onset of anemia, thrombocytopenia and acute kidney injury (30). To further address the similarities and differences between the present case and those previously reported, a comparative summary is presented in Table II. This table contrasts key aspects of presentation, laboratory findings, complications, treatment strategies, and outcomes between our patient and selected cases from the literature (42-45). As demonstrated, the present case shares core features with other pregnancy-associated aHUS presentations, such as rapid postpartum onset of anemia, thrombocytopenia and acute kidney injury, but is distinguished by the presence of severe complications such as diffuse alveolar hemorrhage and ventilator-associated pneumonia, which are rarely documented. These additional complications likely delayed the initiation of targeted therapy, particularly complement blockade, and may have contributed to the persistence of dialysis-dependent renal failure, in contrast to some published cases where earlier intervention was associated with improved hematologic recovery and, in certain instances, partial renal recovery.

Variability in treatment decisions reflects differing clinical scenarios and resource availability, highlighting the importance of individualized, multidisciplinary care (43). In this regard, it is worth noting that the use of eculizumab and other C5 inhibitors, (a humanized monoclonal antibody that inhibits terminal complement activity) has been shown to promote symptom resolution and improve survival, although their impact on renal function is more complex to analyze (44,45). In the case described herein, the complexity of the complications and associated pathologies made it difficult to determine their precise impact; however, a prompter diagnosis may have reduced the morbidity of the patient. Although the present case report highlights key aspects of aHUS management, its findings are limited to a single patient experience and may not be generalizable. Larger case series and systematic studies are required to establish definitive clinical guidelines. Other limitations of the present case report include the absence of genetic testing results and limited long-term follow-up data beyond initial discharge. Future studies are thus required to focus on improving access to genetic diagnostics and on long-term outcomes in pregnancy-associated aHUS. Heightened awareness and timely diagnosis remain crucial to improve patient prognosis and guide therapy. Finally, an important limitation of the present report is the absence of peripheral blood smear images, kidney biopsy histopathology annotations and laboratory trend graphs due to institutional constraints, which would have further supported the diagnosis.

In conclusion, TMAs such as HUS, although rare, confer high morbidity and mortality, often precipitated by pregnancy or puerperal sepsis. The present case report describes a case of postpartum HUS confirmed by renal biopsy following a comprehensive workup including negative antibody and complement tests and ADAMTS13 analysis, which excluded alternative diagnoses. Initial management with plasmapheresis and intravenous steroids yielded favorable outcomes despite in-hospital complications. Given the diagnostic complexity, ruling out differential diagnoses and securing histopathological confirmation remain essential for effective management.

## Figures and Tables

**Table I:** Key laboratory values and clinical milestones following kidney transplantation.

Hospital day/follow-up day	Clinical event/intervention	Hemoglobin (g/dl)	Hematocrit (%)	Platelets (x10³/µl)	Creatinine (mg/dl)	LDH (U/l)	BUN (mg/dl)	Notes
Pre-Tx	Pre-transplant laboratory tests	12.0	37	241	16.2	-	46	Stable baseline
Day 0	Living donor kidney transplant	-	-	-	-	-	-	Warm ischemia 5 min; cold ischemia 48 min
POD 2	Routine laboratory tests	8.4	26	69	1.0	212	10	Albumin 2.2 g/d;; tacrolimus 2.6 ng/ml
POD 4	Routine follow-up	8.6	25	94	0.9	191	9	Tacrolimus 3.4 ng/m;
POD 12	Outpatient control	10.7	34	401	1.0	-	-	Improving counts
2nd visit	Stable	12.0	36	333	1.0	-	13	-
3rd visit	Ultrasound: mild collection	12.7	38	276	1.0	-	14	Upper pole collection 19 cc
Latest visit	Staples and double J removal	-	-	-	1.0	-	-	Asymptomatic

POD, post-operative day; ‘-’ indicates values not available for that date.

**Table II:** Comparative features of the present case and selected previously reported cases of pregnancy-associated atypical hemolytic uremic syndrome.

	Author(s) (Refs.), year of publication				
Feature	Present case	Saad *et al* (30), 2016	Frimat *et al* (42), 2024	Fakhouri (44), 2016	Fakhouri *et al* (45), 2021
Onset	4 Days postpartum	Immediate postpartum	Late pregnancy or postpartum	Postpartum	Postpartum
Main clinical features	Severe anemia, thrombocytopenia, acute kidney injury, diffuse alveolar hemorrhage, ventilator-associated pneumonia	Severe anemia, thrombocytopenia, AKI	TMA signs with anemia, thrombocytopenia, AKI	Anemia, thrombocytopenia, AKI	Anemia, thrombocytopenia, AKI
Laboratory findings	Hb 4.6 g/dl, schistocytes >10/HPF, creatinine 10.6 mg/dl, normal complement levels	Similar hematologic profile, elevated LDH, normal C3/C4	Variable complement abnormalities	Complement abnormalities in subset	Complement abnormalities in subset
Histopathology	TMA in acute and chronic phases, MPGN pattern, severe arteriolonephrosclerosis	Not always performed; when done, TMA lesions	Not always performed; when done, TMA lesions	TMA lesions	TMA lesions
Complications	Diffuse alveolar hemorrhage, ventilator-associated pneumonia	Not reported	Not reported	Not reported	Not reported
Treatment	Broad-spectrum antibiotics, plasma, corticosteroids; delayed immunosuppression; no Eculizumab due to complications and timing	Plasma exchange, supportive care; some cases Eculizumab	Variable: supportive care, plasma exchange, complement blockade	Eculizumab early in course	Eculizumab, some discontinuation
Outcome	Discharged on dialysis, ongoing renal replacement therapy	Variable renal recovery; some dialysis dependence	Variable renal recovery	Hematologic remission; variable renal recovery	Hematologic remission; variable renal recovery

The data from previously reported cases are adapted from previous studies (30,42-45). AKI, acute kidney injury; Hb, hemoglobin; HPF, high-power field; LDH, lactate dehydrogenase; MPGN, membranoproliferative glomerulonephritis; TMA, thrombotic microangiopathy.

## Data Availability

The data generated in the present study may be requested from the corresponding author.
